# Remission from Chronic Anorexia Nervosa With Ketogenic Diet and Ketamine: Case Report

**DOI:** 10.3389/fpsyt.2020.00763

**Published:** 2020-07-30

**Authors:** Barbara Scolnick, Beth Zupec-Kania, Lori Calabrese, Chiye Aoki, Thomas Hildebrandt

**Affiliations:** ^1^Internal Medicine & Addiction Medicine, Waban, MA, United States; ^2^Consultant-Ketogenic Diet Therapy LLC, Elm Grove, WI, United States; ^3^Innovative Psychiatry, South Windsor, CT, United States; ^4^Center for Neural Sciences, New York University, New York, NY, United States; ^5^The Neuroscience Institute, NYU Langone Medical Center, New York, NY, United States; ^6^Center of Excellence in Eating and Weight Disorders, Icahn School of Medicine at Mount Sinai, New York, NY, United States

**Keywords:** anorexia nervosa, ketamine, ketogenic diet, activity-based-anorexia animal model, brain-derived-neurotrophic factor (BDNF)

## Abstract

**Background:**

Chronic anorexia nervosa is a tragic disease with no known effective pharmacological or behavioral treatment. We report the case of a 29 year-old woman who struggled with severe and enduring anorexia nervosa for 15 years, and experienced a complete recovery following a novel treatment of adopting a ketogenic diet followed by ketamine infusions. Her remission has persisted for over 6 months.

**Case Presentation:**

At age 14.5, the patient embarked on an effort to “eat healthy.” She quickly lost control of the dieting, developed associated compulsions and obsessions about food, body dissatisfaction, emotional lability, and lost nearly 13.6 kilograms (30 pounds). She was hospitalized for 6 weeks, and while she regained some weight, she did not attain full weight restoration. For 15 years, she continued to eat in a restrictive manner, exercise compulsively, and have intermittent periods of alcohol dependence. Nevertheless, she always hoped to get well, and at age 29, she began a novel treatment for anorexia nervosa.

**Conclusions:**

This is the first report of a ketogenic diet used specifically for the treatment of anorexia nervosa, followed by a short series of titrated IV ketamine infusions leading to complete remission of severe and enduring anorexia nervosa, with weight restoration, and sustained cessation of cognitive and behavioral symptoms, for 6 months. Although these treatments were used sequentially the relationship between these modalities, and possible synergy, is unclear, and deserves further study. Complete and sustained remission of chronic anorexia nervosa is quite rare, and the novel use of a ketogenic diet and IV ketamine treatment in this potentially lethal condition suggests avenues for further research, and hope for patients and their families.

## Introduction

Anorexia nervosa (AN) is a severe disorder characterized by self-starvation, hyperactivity, anxiety, body dissatisfaction, and emotional dysregulation that usually begins in early puberty, and affects females more frequently than males. When weight restoration is the only criteria measured, rates of 60% recovery are often noted, but when cognitive recovery is included, the results are more discouraging (1–3). Severe and enduring anorexia nervosa refers to cases that have persisted for at least 3 years, and are resistant to intervention. It is a tragic disorder with no consistently effective pharmacological or behavioral treatments. The mortality rate is estimated to be ten times higher than that of the general female population, due to medical complications of self-starvation, and suicide (4). We report a case of severe and chronic AN treated successfully by adopting a ketogenic (KG) diet for 3 months followed by a series of intravenous ketamine infusions.

Emerging data suggest that disturbances in lipid signaling may underlay some of the core pathology of anorexia nervosa (5–7) and that chronic starvation may promote a type of metabolic hibernation in patients (8) that requires normalization of lipid signals to resume premorbid metabolic and central nervous system functions. A KG diet is a high fat, low carbohydrate medical diet, that leads to increased fatty acid oxidation for cellular energy, which in turn results in increased ketone body production. Ketones are produced in the liver whenever fat metabolism increases over carbohydrate metabolism; such as fasting, post-exercise, neonatal period, and KG diet, as well as during pathological states such as starvation or uncontrolled diabetes mellitus (9). Ketones easily pass the blood brain barrier and become the brains’ major energy source. This change in metabolic fuel from glucose to ketones leads to a myriad of changes in central nervous system neurotransmitters such as adenosine and gamma-aminobutyric acid (GABA), as well as neuropeptides such as leptin, adiponectin, and growth hormone-releasing peptides (10). As far as we know, this is the first publication describing the use of a KG diet for patients with AN.

In addition to dietary disturbances and potential metabolic disturbances, psychiatric symptoms such as anxiety, obsessive-compulsive disorder, depression, and addiction are frequently observed among individuals with AN (11). Ketamine has emerged as a novel treatment for treatment-resistant mood disorders, particularly those with suicidal ideation (12, 13). As far as we are aware, there is only one published study of ketamine infusions for AN, and it was encouraging (14).

We report the case of a 29 year-old woman, with chronic severe AN who had an excellent response to adopting a ketogenic diet and undergoing ketamine infusions.

## Case Presentation and Clinical Assessment

The patient was a 29 years old single woman, diagnosed with AN at age 14.5, and intermittent alcohol dependence beginning at age 18. She was not in active psychiatric treatment, although she saw a supportive therapist episodically and attended Alcoholic Anonymous meetings at least once/month. She had been abstinent for 15 months, and was eating with many restrictions—preferring to eat by herself and use a napkin rather than a plate, eating only ice cream for days at a time, and excessively using non-nutritive low calorie sweeteners. She exercised daily and compulsively, usually spending 2 h at the gym. Her weight had been stable at 56.8 kg (125 lbs) for several years. Recognizing she was living a very limited life, she decided to try a novel treatment, and worked closely with an internist who was knowledgeable about eating disorders, who is the first author.

The patient was the product of a normal pregnancy and delivery and raised in an upper middle class home by parents who are both attorneys. Her older sister was very concerned with her own appearance. Family history was significant for a paternal grandmother who was obsessed with “being very slender” all her life, though was never given a psychiatric diagnosis. The patient’s mother was diagnosed with an anxiety disorder and was prescribed citalopram.

At an early age, the patient was identified as athletic, artistic, and excelled academically. Her parents noted that she had “compulsive tendencies’ such as “grinding erasers” and wanting her possessions to be lined up symmetrically, but this never reached the level of clinical concern.

At age 14.5, while at a sleep-away camp, she began a diet, with the aim of “eating healthier” with other girls in her bunk. When summer ended, she continued her diet. Her parents slowly became aware of a change in her mood and behavior, from a happy child to a sullen and angry teenager. They initially attributed this to the “onset of adolescence” but when her weight loss became more obvious, she was evaluated by her pediatrician, and was referred to an outpatient eating disorder clinic, where she ate 3 dinners/week. She also had weekly individual supportive therapy, and weekly meetings with a nutritionist. Her parents also saw a therapist separately. Her weights are summarized on **Table 1**; prior to illness she weighed 61.1 kg (136 lbs); height 1.62 meters (5’4”) with a Body Mass Index (BMI) of 23.3.

**Table 1:** Weights over the years.

Age	Circumstance	Weight (kg)	Weight (lb)	Body Mass Index
14.5	Prior to illness	61.7	136	23.3
16	Acute AN—outpatient	42.6	94	16.1
17	Hospitalization	50.8–54.4	112–120	19.2–20.6
18-21	Community college	52.6–56.7	116–125	19.9–21.5
21-22	Out of state college	46.2–52.1	102–115	17.5–19.7
22-29	Working in wilderness program; starting graduate school	53.5–56.7	118–125	20.3–21.5
29	Immediately prior to starting KG diet	57.2	126	21.6
30	Ketamine Injections over 2 weeks	58.9	130	22.3
30	3 months post treatment; in full remission	61.7	136	23.3

Twelve months into the course of her illness, she had a grand mal seizure. At the time, her weight was 43.5 kg (96 lbs) with BMI of 16.5, and her treatment was changed to incorporate Maudsley treatment in which the parents are actively taught to re-fed the child (15). She was resistant, and meals were rarely successful; after 6 months of out patient treatment she was hospitalized for 15 days followed by 30 days of “partial hospitalization.” On admission to the inpatient unit, her weight was 50.8 kg (112 lbs.) with a BMI of 19.2. Her pulse was 80 sitting, rising to 103 standing, and her blood pressure was 94/60 sitting; and 110/82 standing. The remainder of the exam was normal as were her electrolytes, and kidney and renal function tests, and complete blood count, MRI, EEG, and EKG. A bone density exam showed mild osteopenia of the hip and spine. She had had a normal menarche age 13, but had been amenorrheic for 7 months. She denied binging, purging, laxative, or diuretic abuse and was given the diagnosis of anorexia nervosa restricting type. She was discharged to home after attaining a weight of 54.4 kg (120 lbs.) with a BMI of 20.6.

She returned to high school, yet she never regained friendships she had prior to the hospitalization, and remained dismissive of her parents’ efforts to supervise her eating. Her weight generally hovered near 54.5 kg (120 lbs.). She started college several times, and dropped out several times, but eventually graduated from an excellent university. Over the next 8 years, she had three month-long admissions to alcohol & drug rehabilitation programs. She worked as a recovery coach in several wilderness rehabilitation programs, and began graduate school.

The only medications she was prescribed were lamotrigine for six months following the seizure, and lisexamfetamine dimesylate (Vyvanse).

At age 29, on physical exam, she appeared fit and well. Her BP was 120/72 and pulse 50, without orthostatic changes, and her weight was 56.7 kg. (125 lbs). Her physical exam was normal, as was her complete blood count, electrolytes, liver and kidney function tests. She was coached by an experienced KG nutritionist on ways to prepare high fat, low carbohydrate meals and smoothies, and encouraged to eliminate non-nutritive sweeteners. The goal was to induce a stable degree of ketosis, following a liberal KG diet with a 2:1 to 1:1 ratio of fats: carbohydrates + protein in grams. While we anticipated it might be difficult for her to eat fatty foods, as it is known that individuals with AN avoid high calorie foods (16), she found it relatively easy.

- “The whole time I was anorexic, I ate my safe foods, and there were not many: Lots of Splenda®, frozen yogurt, zucchini, eggplant and coffee. I only ate such a ridiculously limited amount of food, that the logistics of switching to keto were not that hard - when you think you want x, have y instead. If you have three x’s and that’s it, then have three y’s, and for me the ketos were bacon, eggs, cheese avocado, Medium Chain Triglyceride (MCT) oil”

Initially, she varied between strictly following the KG diet and being lax, but gradually found she was gaining insights into the anorexic “voice”, and it became easier to follow the diet and remain in ketosis.

- “Until now (KG), I ate sweetener with everything. I used to put Splenda on broccoli.”
- “I now consider myself to have a sugar allergy. When I eat avocados or bacon and eggs, the “voice” is softer, if I eat sugar I feel euphoric, but the euphoria has feelings of panic.”

She measured breath acetone levels with a portable breath monitor, 2–3 times/week. Breath acetone levels are scored qualitatively from 0 to 6, and she was at 2 or 3 (mild-medium range) when she was on the KG diet.

After 3 months on the diet, she reported she was “30-40% better, but was still troubled by anorexic compulsions.” She was referred for ketamine infusions. She underwent comprehensive evaluation, and met DSM-V criteria for anorexia nervosa, restricting type, and major depressive disorder, recurrent, moderately severe, with Patient Health Questionnaire-9 (PHQ-9) of 13 (17). She began treatment with racemic IV ketamine at dose of 0.75 mg/kg (42.68 mg), in 30 cc 0.9% normal saline infused over 45 min in a private room with dimmed lights, sound machine, using an eye mask. Pulse, blood pressure, respiration, O2 saturation, and cardiac waveform were monitored continuously throughout her infusion and showed expected variations in pulse and blood pressure which did not require intervention. Dissociation was present, as evidenced by patient report of feeling “out of my body” and “seeing things in layers,” and clinical observations. She displayed decreased verbal fluency and mild 1-2 beat end-gaze nystagmus during treatment, which resolved completely. She developed mild nausea following her first infusion. There were no other reported or observed side effects during treatment. All treatment-emergent side effects dissipated within 20 min. Within one hour of her first infusion, she felt the anorexic voice was weakening, and she had more ability to “be herself.” PHQ-9 dropped to 6. She then had three more IV ketamine infusions over the next fourteen days, at doses titrated to 1.0 mg/kg, 1.1 mg/kg, and 1.2 mg/kg to achieve and maintain dissociation during infusion. Each infusion was preceded by ondansetron 4 g sublingual and she denied nausea during or after these infusions. Her response was dramatic and occurred after the 4^th^ infusion.

- “I know this sounds ridiculous, but I am no longer anorexic. I had so many rules I didn’t even know them. But they are gone. I can exercise because it feels good. It isn’t that I have to. I can stop when I want to.”

Notably, her PHQ-9 dropped to 6 after her first treatment, and to 2 following her third infusion.

### Follow Up

She has continued to feel free from the intrusive AN obsessive thoughts, rigid AN rules, and compulsive behaviors, and to explore a fuller life, and is now over 6 months post ketamine infusion. She continues to eat a KG diet and her weight has inched up to 61.3 kilograms (136 pounds), the weight when the disease took hold at age 14.5. When she weighs herself now she notes:

- “I really didn’t know how I would feel getting on a scale and looking at my weight. Now, I am so relieved. I think I do not CARE about the number because I can SEE myself now in the mirror, so the number on the scale is ‘like’ ONE source of information, but not the ONLY one. I think 136 pounds is fine and I LOVE the way I look”,

Following treatment, she sees a therapist weekly for supportive psychotherapy, although during the COVID-19 pandemic visits have occurred by telemedicine. She checks her own weight every few weeks when she visits her cousin’s apartment where there is a scale, and it has been stable at her premorbid weight of 61.8 kg (136 lbs).

### Patient Perspective

“All these years, I was still in there watching, waiting, and hoping to escape. Every single interaction now is a new freedom.”

She is very supportive of this publication and hopes this treatment will be studied to determine if it is effective for other patients who suffer, as she did.

## Discussion

This is a striking case report of an unusual combination of a ketogenic diet and a short series of titrated ketamine infusions in a patient with severe chronic AN of 15 years – modalities that have a rational medical justification, but have never been used clinically for this disorder, or in this patient population. This resulted in a surprising and long lasting complete remission which has continued despite the stressors of living in New York City in the midst of the COVID-19 pandemic. The strength of this case report is that it offers an innovative approach to chronic AN, a disorder which is exceedingly resistant to treatment and associated with an increased risk of death.

KG diets for AN have been roundly dismissed by eating disorder experts, and with the exception of an early study in 1998, ketamine has not been reported as effective in AN. Thus, it is critical to consider the context of how this treatment sequence evolved for this patient.

The impetus for this treatment arose when the patient was presented with an etiological theory of AN, known as Adaptation to Flee Famine Hypothesis, which frames the genesis of AN as metabolic rather than psychological (18). This resonated deeply with the patient, as she often had felt she was battling invisible forces. Seeing her symptoms as the result of distorted physiological signals and offering a metabolic solution was powerfully motivating. Explaining that there was an animal model for AN that replicated many of its symptoms, was also empowering, as it countered the narrative that she was somehow “choosing” this disorder in order to exert “control” over her life. These psycho-social constructs, which she had heard many times in rehabilitation centers, had never rung true for her, and had also burdened her with guilt for “causing her own illness.”

The Adaptation to Flee Famine Hypothesis posits that it would have been evolutionarily advantageous to a nomadic tribe if some members acted uniquely when presented with a state of food scarcity or famine. If these individuals could deny hunger, dismiss their obvious body inanition, act as if they were just fine, and engage in hyperactive running long distances, they might lead the tribe to better hunting grounds. These are precisely the behaviors seen in adolescents with AN.

The activity-based-anorexia rodent model involves housing a rodent in a cage with a running wheel, and decreasing the food supply slightly (19). Some animals, often female and adolescent, will increase running and reduce eating drastically, thus replicating aspects of human AN. The model requires both stimuli of decreased food supply plus unlimited access to a running wheel.

Advising the patient to adopt a KG diet was prompted by two factors. First, two studies employing an activity-based-anorexia model demonstrated that a KG diet rescued the animals from self-starvation (20); and a high-fat diet (although not ketogenic) prevented the animals’ self starvation (21). Secondly, the KG diet has an impressive record of safety and efficacy for treating seizure disorders in children, and has been used continuously since the 1920s (22). It is an emerging therapy for treatment of other neurological and psychiatric disorders such as Parkinson’s, early Alzheimer’s, and bipolar disorder (23). Thus, since it is generally safe and effective for other neurological disorders, it might also prove effective for AN, especially since AN has been shown to be genetically linked with abnormalities in fat metabolism (6).

There are known side effects of the KG diet which are occasionally observed in patients treated for seizures. These include weight loss, constipation, nephrolithiasis, and hypoglycemia. These effects are seen most often in children who are on multiple anti-seizure medications and are often physically compromised. Fortunately, this patient did not experience any of these symptoms and her weight increased as she ate with more freedom. The patient was at a stable weight and the only medication was lisexamfetamine dimesylate (Vyvanse). Furthermore, a more rigid ketogenic diet of 4:1 fat: protein plus carbohydrate diet is often employed for patients with seizures, while the diet employed in this case was a more liberal diet of 2:1 or 1:1 which may have mitigated some of the possible side effects of the KG diet.

It is unknown whether the positive results from the KG diet arm of the treatment were due to adopting a KG diet, or if simply adopting a high fat diet without the carbohydrate restriction required for ketosis would have been effective. We believe it was the ketogenic nature of the diet because the biochemical changes seen with ketosis are different from those seen with increased fat. A recent article testing a ketogenic diet in humans, and also transplanting the microbiome into mice, noted that the microbiome responds differently to a moderate fat diet, a high fat diet that is not ketogenic, and a high fat/low carbohydrate that is ketogenic (24). This is an area of active research.

If the patient had been pleased with the results of the KG diet alone, the treatment would have stopped at that point. She reported the symptoms of AN were “30-40% improved but she was still troubled with anorexic compulsions. Thus the second “arm” of the treatment began—ketamine infusions. The addition of ketamine treatment in this case was also based on studies on the activity-based-anorexia model, plus one very interesting, and overlooked case series report from 1998.

Experiments with the activity-based-anorexia model found that individual rodents vulnerability to self-starvation correlated with levels of N-methyl-D-aspartate (NMDA) glutamine receptors in the dorsal hippocampus (19). Since ketamine is a well known antagonist to NMDA glutamine receptors, an elegant experiment examined whether injection of ketamine on day 2 when the animal had started to self-starve and hyper-exercise could alter the behavior (25). As predicted, those treated with ketamine ate more, exercised less, and were resilient to the self-starvation when they were re-exposed to the paradigm, compared to those treated with placebo.

Very similar reasoning was described for the rationale of a study from Cambridge, England, published in 1998 in which Mills et al. treated 15 patients with chronic severe AN with a series of ketamine infusions (14). Significant prolonged improvement was reported in 9 patients. Some of the responses resembled the dramatic changes noted in this case report.

It is difficult to determine the doses of ketamine administered in this report because the authors describe that infusions were delivered at 20 mg/h for 10 h using doses “similar to that used to produce analgesia” but exact doses were not stated. Furthermore all patients in this series were prescribed an opiod blocker to “ensure they remained conscious”.

We approached the ketamine arm of the treatment cautiously because the patient had struggled with alcohol dependence, substance abuse is common in patients with AN, and ketamine is a drug that is recreationally abused. A recent study, pooling data from 5 clinical trials found no evidence of serious side effects from a single infusion of ketamine for clinical depression, and ketamine was not associated with increased abuse (26). Many patients reported feeling “loopy” or “dissociative” during the infusion, but this passed rapidly. While this study is promising, racemic ketamine infusions for psychiatric disorders remains off-label, and issues of optimal dose, duration, titration, and number of infusions needs further research. In AN, these questions deserve further investigation and warrant controlled trials. This patient did indeed experience dissociation during her infusions, which she found was part of its therapeutic effect.

Although the efficacy of KG diet for seizure disorders, and ketamine infusion for severe treatment resistant depression has been clearly demonstrated, the mechanisms of actions of both agents are not well understood. It is notable that both ketamine and KG diet have been shown to intimately affect brain-derived neurotrophic factor (BDNF), gamma-Aminobutyric acid (GABA), and N-methyl-D-aspartate (NMDA) glutamate receptors which are the same molecules affected in the activity- based-anorexia model (27, 28). It is possible that the KG diet and IV ketamine act synergistically, with the KG diet “priming” the response to ketamine. This requires further study.

There are several important limitations to this report and to the generalizability of the findings. Most importantly, this is a single case report and further study is needed. Secondly, standardized eating disorder rating scales were not used to monitor progress. This is because this is a report about a clinical experience with a patient used to monitoring her own eating and activity levels by journaling; it was not organized as a clinical trial. There are no eating disorder scales that have been validated for used with ketamine treatment. Third, this treatment paradigm essentially involved three modalities, and not just two treatment arms: first the patients adoption and reconsideration of AN as a disorder whose symptoms are predominately metabolically driven, which was an enormous paradigm shift for her; secondly adopting the KG diet and contextualizing ketosis as helpful for her brain rather than as a weight loss or weight control method for her body; and third IV ketamine infusions at a dose and titration schedule not previously reported. The fact that this sequential treatment resulted in complete and sustained remission of this patient’s severe AN thoughts and behaviors was unexpected. Furthermore, her remission has persisted despite extreme stressors, social isolation, work restrictions, and treatment disruptions during the COVID-19 pandemic in NYC. At his point it is not possible to state which modality was essential, or which had the most important therapeutic benefit, if both ketogenesis and ketamine are necessary and synergistic, or if the treatment would have been as effective if the KG diet were initiated with ketamine when ketamine infusions began instead of preceding them by several months. It is possible that ketamine infusions alone would have been effective. Future studies are necessary to address these questions, and to address optimal KG diet ratios and optimization of ketamine dose, duration, and frequency.

## Conclusions

There remain many unanswered questions about the generalizability of these findings to other patients with AN. Yet, the medical plausibility of this treatment and the dire prognosis of chronic anorexia nervosa, suggest that both the KG diet and ketamine treatment warrant further study in this patient population towards the goals of restoring weight, normalizing cognitive and emotional functioning, and improving quality of life. It is hoped that this report will stimulate this research.

## Data Availability Statement

The original contributions presented in the study are included in the article/supplementary material; further inquiries can be directed to the corresponding author.

## Ethics Statement

Ethical review and approval was not required for the study on human participants in accordance with the local legislation and institutional requirements. The patient provided her written informed consent to participate in this study. Written informed consent was obtained from the individual, for the publication of any potentially identifiable images or data included in this article.

## Author Contributions

All authors contributed to the article and approved the submitted version. BS orchestrated the treatment and wrote the first draft of paper. BK coached the patient on the ketogenic die and edited the paper. LC performed a comprehensive evaluation of the patient, treated the patient with ketamine infusions, and edited the paper. CA performed the studies on activity-based-anorexia, kept the authors informed of her studies, and edited the paper. TH was the primary medical provider 15 years ago when the patient first became ill, continued to consult regarding her care over the years, and edited the paper.

## Conflict of Interest

Author BZ-K was employed by the company Ketogenic Diet Therapy LLC.

The remaining authors declare that the research was conducted in the absence of any commercial or financial relationships that could be construed as a potential conflict of interest.
